# Inflammation in atherosclerotic cardiovascular disease

**DOI:** 10.12688/f1000research.18901.1

**Published:** 2019-08-09

**Authors:** Prediman K. Shah, Dalgisio Lecis

**Affiliations:** 1 Helga and Walter Oppenheimer Atherosclerosis Research Center, Smidt Heart Institute, Cedars Sinai Medical Center, Los Angeles, CA, USA; 2 Department of Cardiovascular Medicine, "Tor Vergata" University of Rome, Rome, Italy

**Keywords:** Atherosclerosis, Inflammation, thrombosis

## Abstract

Atherosclerotic cardiovascular disease is a leading cause of death and morbidity globally. Over the past several years, arterial inflammation has been implicated in the pathophysiology of athero-thrombosis, substantially confirming what pathologist Rudolf Virchow had observed in the 19th century. Lipid lowering, lifestyle changes, and modification of other risk factors have reduced cardiovascular complications of athero-thrombosis, but a substantial residual risk remains. In view of the pathogenic role of inflammation in athero-thrombosis, directly targeting inflammation has emerged as an additional potential therapeutic option; and some early promising results have been suggested by the Canakinumab Anti-inflammatory Thrombosis Outcome Study (CANTOS), in which canakinumab, a fully human monoclonal antibody targeting the pro-inflammatory and pro-atherogenic cytokine interleukin 1 beta, was shown to reduce cardiovascular events.

## Introduction

Cardiovascular disease from atherosclerosis manifests as acute and chronic ischemic syndromes such as acute coronary syndromes, angina pectoris, claudication, ischemic strokes, congestive heart failure, and sudden and non-sudden cardiac death ^1– 4^ Atherosclerosis consists of build-up of plaque inside the intima of medium and large arteries, leading to chronic luminal narrowing or disruption of the plaque surface (plaque rupture or superficial erosion) with superimposed thrombosis and a subacute or acute luminal compromise. Most of the acute and life-threatening manifestations of atherosclerosis result from plaque disruption and thrombosis ^1– 4^.

## Pathophysiology of atherosclerosis

Atherosclerotic plaques contain a variable mix of lipids, smooth muscle cells, extracellular matrix, calcium, and components of the immune system (both from innate and adaptive immunity) such as macrophages, dendritic cells, mast cells, natural killer cells, and T cells. In addition, increased plaque neovascularization and intraplaque hemorrhage are features of atherosclerotic plaques. Although the precise mechanism of athero-thrombosis remains incompletely understood, a number of risk factors that increase the likelihood of atherogenesis have been identified: these include dyslipidemia with elevated apolipoprotein B (apoB) 100–containing lipoproteins, low levels of high-density lipoprotein (HDL), hypertension, diabetes, smoking, central obesity and metabolic syndrome, advanced age, menopause, genetic factors and family history of premature coronary disease, chronic immune-inflammatory conditions (such as psoriasis, rheumatoid arthritis [RA], systemic lupus erythematosus, HIV, and Kawasaki’s syndrome), chronic infections, and radiation exposure ^1– 5^. Cardiovascular disease is now recognized as a major cause of premature mortality among patients with autoimmune chronic inflammatory conditions, and there is an urgent need to identify those who are at risk of cardiovascular ischemic events in order to optimize prevention and therapeutic intervention ^6^. In this regard, several clinical trials showed that methotrexate use is associated with a reduced risk of cardiovascular events in patients with RA. This suggests that reducing the inflammation in RA by using methotrexate not only improves disease-specific outcomes but also may reduce collateral damage such as atherosclerosis ^7, 8^.

## Key role of lipids in atherogenesis

It is generally agreed that lipids play a key role in the initiation of atherosclerosis. Experimental observations have suggested that one of the earliest events in atherogenesis is the entry of atherogenic (apoB 100–containing) lipoproteins into the sub-endothelial space, where they interact with extracellular matrix components, leading to trapping of lipoproteins with subsequent aggregation and oxidative modification and then to generation of pro-inflammatory lipids ^9, 10^. These pro-inflammatory lipids lead to endothelial dysfunction manifesting with increased adhesivity of endothelium to circulating mononuclear cells which then are recruited into the sub-endothelium aided by the local production of inflammatory cytokines. Monocytes in the sub-endothelium mature into macrophages expressing scavenger receptors through which the lipids are engulfed, turning monocyte-derived macrophages into foam cells. Some foam cells are also derived from vascular smooth muscle cells. Monocyte-macrophages in the lesion secrete mediators that also recruit smooth muscle cells from the media; these smooth muscle cells migrate, proliferate, and secrete matrix proteins, contributing to build-up of plaque; some macrophages and dendritic cells present neoantigens to the T cells, creating a pro-inflammatory adaptive immune response that perpetuates inflammation in the plaque ^1^.

## Critical role of inflammation in hyperlipidemia-induced atherogenesis

**Figure 1.**
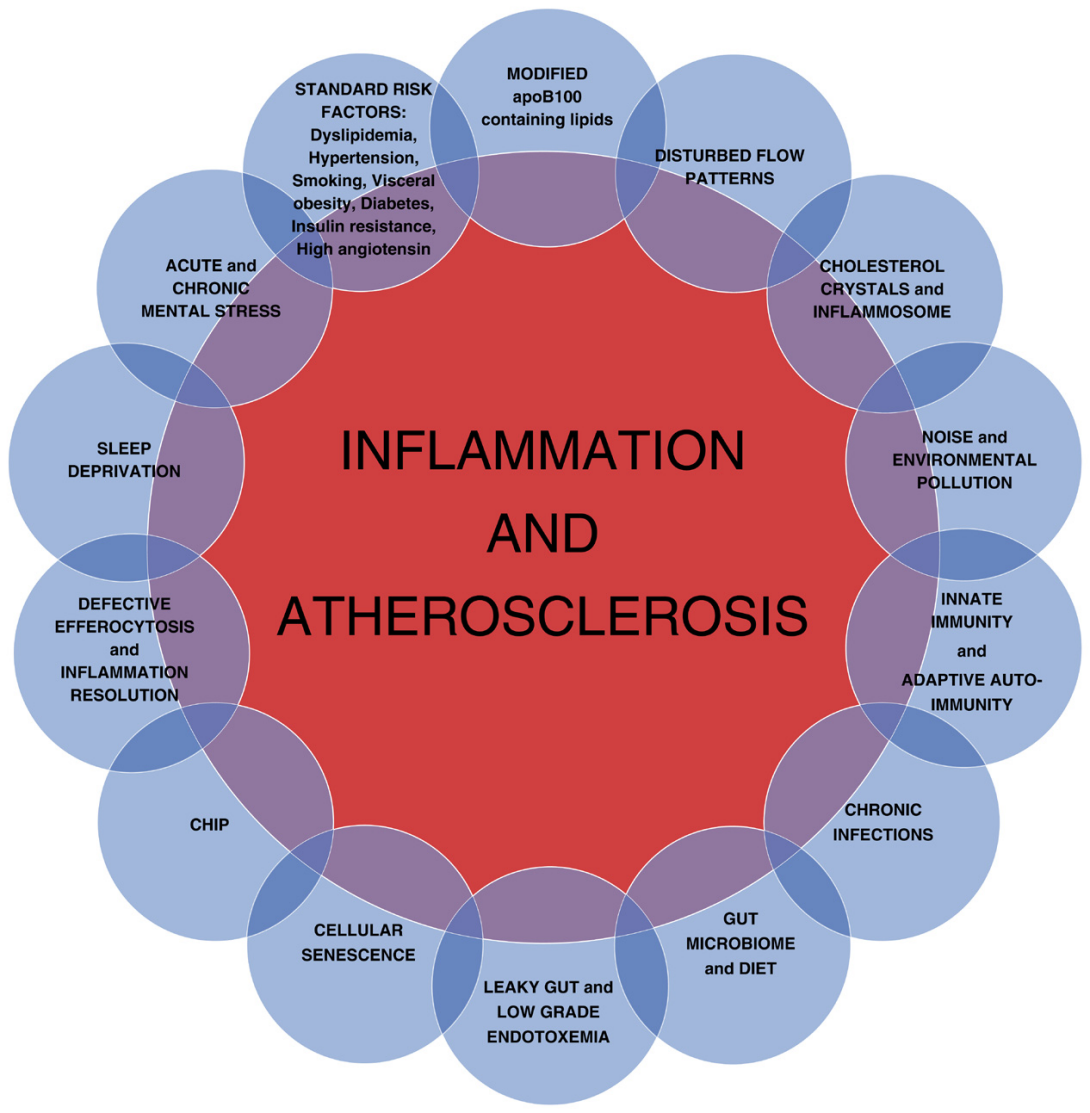
Different pathways for inflammation in athrosclerosis are depicted in this figure. (CHIP, clonal hematopoetic mutations of indeterminate potential).

Experimental studies and many clinical observations have shown that hyperlipidemia is essential but not sufficient to produce atherosclerosis unless there is inflammation as well. Inflammation was, in fact, implicated in atherosclerosis by Virchow as far back as in 1858 ^11^. Many cytokines and chemokines are involved in the development and progression of the atherosclerotic plaque. Some of them, such as colony-stimulating factor-1 (CSF-1) and monocyte chemoattractant protein-1 (MCP-1), whose partial or complete deletion dramatically reduces atherosclerosis in murine models despite severe hyperlipidemia, are important in the initial phases of plaque formation ^12^. Hyperlipidemia activates innate immunity by activating Toll-like receptor 2 (TLR-2) and TLR-4 pathways, leading to activation of inflammatory and pro-atherogenic genes in macrophages and endothelial cells ^12^. Disruption of lipid-induced innate immune signaling reduces atherosclerosis in hyperlipidemic murine models ^12^. Given the multifactorial nature of cardiovascular disease and the complexity of the inflammation pathways involved in atherosclerotic plaque development (as shown in Figure 1), the implications of findings in hyperlipidemic mice have to be carefully assessed when considering humans. In addition to apoB 100–containing lipoproteins, HDL may become pro-inflammatory and pro-atherogenic when it undergoes chemical modification by macrophage-derived myeloperoxidase or mast cell–derived proteases through acquisition of pro-inflammatory mediators (such as serum amyloid A and symmetrical dimethyl arginine) or loss of anti-inflammatory mediators (such as clusterin, paraoxonase, and apoA-1), creating a dysfunctional form of HDL which promotes atherosclerosis ^13^. Furthermore, mast cell–derived neutral proteases neutralize some of the critical anti-atherogenic functions of HDL. Thus, they degrade the pre-beta–HDL fraction, thereby blocking the ABCA1-dependent cholesterol efflux from foam cells ^14^. Moreover, the anti-inflammatory functions of apoA-1 on endothelial cells are lost upon its C-terminal cleavage by the human mast cell neutral protease chymase ^15^.

Thus, a large body of evidence implicates inflammation in the initiation and progression of atherosclerosis ^1– 5, 12^. Inflammation has also been implicated in outward remodeling that occurs with plaque formation, intraplaque neovascularization and plaque hemorrhage, matrix depletion with thinning of the fibrous cap through increased matrix proteolysis mediated by matrix-degrading enzymes, and eventually disruption of the fibrous cap leading to thrombosis ^1– 5, 12^. Plaque thrombogenicity has also been attributed to macrophage-derived tissue factor within the plaque ^1– 5, 12^. Thus, inflammation plays an important role at multiple steps in the evolution of athero-thrombosis.

## Do other factors contribute to inflammation in atherosclerosis?

### Local shear stress and flow dynamics

Atherosclerotic plaques tend to preferentially form at sites of low or oscillating shear stress, such as branch points and curvatures where flow patterns are disturbed; these disturbed flow patterns appear to promote entry of atherogenic lipoproteins by increasing their residence time; in addition, such disturbed flows promote a pro-inflammatory endothelial phenotype that is orchestrated by flow-sensitive transcription factors such as KLF2; inhibition of KLF2 by low shear stress promotes a pro-inflammatory phenotype contributing to atherogenesis and plaque inflammation ^16^. Murine studies have shown that athero-prone sites of normolipidemic mouse aorta contain cellular components priming these sites for enhanced inflammatory responses ^17^.

### Cholesterol crystals and inflammasome activation

Cholesterol crystals, frequently present in atherosclerotic plaques, can activate the NLRP3 inflammasome pathway to induce secretion of pro-inflammatory and atherogenic cytokines like interleukin 1 beta (IL-1β) and IL-18 ^18– 22^. Activation of NLRP3 inflammasome requires a priming signal which can be provided by neutrophil-derived extracellular traps and by oxidized lipids followed by the second signal provided by cholesterol crystals ^19^. Mitochondrial damage and dysfunction can also play important roles in activating NLRP3 inflammasome ^21, 22^.

### Integrated stress response, inflammation, and atherosclerosis

Dyslipidemia, especially exposure to saturated fatty acids, induces endoplasmic reticulum stress, mitochondrial oxidative stress, and elF2 alpha phosphorylation which hyperactivates an integrated stress response (ISR), which in turn activates a local and systemic inflammatory response through activation of NLRP3 inflammasome: this process contributes to atherogenesis since inhibition of ISR at different nodes reduces atherosclerosis in hyperlipidemic murine models ^22^. Small-molecule ISR inhibitors could emerge as important anti-inflammatory agents for atherosclerosis and other chronic inflammatory conditions ^22, 23^.

### Visceral obesity, insulin resistance, and type II diabetes mellitus

Several studies have shown that visceral adiposity is associated with organ-specific inflammation involving the adipose tissue, liver, pancreas, and arterial wall; visceral adipose tissue of diet-induced obese mice was demonstrated to be rich in T cells secreting interferon gamma (IFN-γ) at higher levels than lean controls. This inflammation is associated with insulin resistance and metabolic syndrome, eventually contributing to the development of type II diabetes mellitus ^24– 28^. Diabetes-associated dyslipidemia and pro-inflammatory state contribute to enhanced atherogenesis observed with diabetes ^24– 28^.

## Smoking and environmental pollution

Active smoking and passive exposure to smoking are associated with increased atherogenesis and risk of acute vaso-occlusive cardiovascular events mediated in part by pro-inflammatory and pro-thrombotic effects of smoke exposure ^29^, which are associated in part with perturbations on lipid metabolism ^30^. Similarly, environmental pollutants may induce inflammation, enhancing risk of cardiovascular disease ^29^. The underlying mechanism in both cases may be due to the increased production of reactive oxygen species (ROS) exceeding the endogenous antioxidant capacity associated with an increase in the markers of inflammation ^31, 32^.

## Hypertension and inflammation

Recent studies have implicated oxidative stress and innate and adaptive immunity in hypertension and hypertension-related end organ damage. Angiotensin II, salt retention, or increased mineralocorticoid activity activates innate immunity that precipitates or aggravates hypertension ^33^. These hypertensive stimuli also induce oxidative stress in antigen-presenting cells which lead to neoantigen formation ^33^. These neoantigens in turn lead to an adaptive immune response which can further damage end organs such as the kidney ^33^. Several experimental studies have implicated T helper 17 (Th17) cells and their cytokines in inflammation in hypertension, and IL-10 has a counterbalancing role by producing regulatory T (Treg) cells ^33^.

## Adaptive autoimmunity and inflammation in atherosclerosis

An adaptive immune response to autoantigens, both humoral and cell-mediated, exists in animal models of atherosclerosis and in human subjects ^1, 34, 35^. T-cell activation occurs upon presentation of the antigen in the setting of an inflammatory state, resulting in clonal proliferation and in the differentiation of CD4 ^+^ T cells to Th1, Th2, or Th17 phenotype, depending on the cytokines secreted by the antigen-presenting cells ^1, 34, 35^. Both a pro-atherogenic inflammatory immune response mediated by Th1 and possibly Th17 and B-cell subsets and an athero-protective anti-inflammatory immune response mediated by Treg cells and B1 cells have been shown to exist and modulate atherosclerosis ^1, 34, 35^. Autoantigens that have been identified include antigens derived from both the protein and lipid components of apoB 100–containing lipoproteins, heat shock protein 60 (HSP 60), and beta glycoprotein ^1, 34, 35^. These observations have led to the concept of immunomodulatory therapies for atherosclerosis, which are being developed in various laboratories ^34, 35^.

### Infections, atherosclerosis, and cardiovascular events

Several studies have implicated infections to either atherogenesis or precipitation of acute cardiovascular events ^5, 36, 37^. These infections include influenza, gingivitis, urinary tract infections, skin infections, HIV, pneumonia, and *Helicobacter pylori* infections ^5, 36, 37^. The link between infection and atherosclerosis has been attributed to direct infection of the vessel wall ( *Chlamydia pneumoniae*), indirect effects involving molecular mimicry, or systemic pro-inflammatory effects ^5, 37^. However, a number of large-scale randomized clinical trials targeting *C. pneumoniae* with antibiotics failed to reduce cardiovascular events ^5^. On the other hand, influenza vaccination has been shown to reduce cardiovascular events in a limited number of randomized clinical trials and in many observational studies ^5^. The cardioprotective effects of pneumococcal vaccines have not been as persuasively demonstrated ^5^.

### Diet and gut microbe interaction

In recent years, gut microflora has been implicated in the pathogenesis of a number of diseases, including obesity, diabetes, hypertension, atherosclerosis–thrombosis, and neurodegenerative diseases ^38^. Several studies have suggested that certain dietary constituents such as phosphatidylcholine, choline, and carnitine are acted upon by enzyme trimethylamine lyase (TMA lyase) produced by gut microbes such as Clostridia, Shigella, Proteus, and Aerobacter to generate TMA which is converted into trimethylamine oxide (TMAO) by hepatic flavin mono-oxygenases. TMAO enhances foam cell formation by upregulating macrophage scavenger receptors which may contribute to its pro-atherogenic effects ^38^. In addition, TMAO enhances platelet activity and predisposes patients to thrombosis ^38^.

In human subjects, circulating TMAO levels correlate with the presence of coronary artery disease and the future risk of athero-thrombotic cardiovascular events ^38^. TMAO also has been shown to contribute to enhanced atherogenesis in murine models and enhanced platelet aggregation ^38^. In murine models, antibiotics directed at gut microflora reduce atherogenesis ^38^. Inhibition of TMA-generating microbial enzymes by dimethylbetane also reduces murine atherosclerosis ^38^. Consumption of a Mediterranean-type diet is also associated with lower circulating levels of TMAO ^38^ and this may account for the anti-inflammatory and health-promoting effects of a Mediterranean diet. Recently developed, non-toxic potent inhibitors of gut microbial TMA lyase (halomethylcholines) were shown to markedly inhibit platelet reactivity and thrombosis ^38^.

### Leaky gut and low-grade endotoxemia

A number of studies have shown that low-grade endotoxemia due to leaky gut is present in human subjects under certain conditions and that, in animal models, such low-grade endotoxemia has pro-inflammatory effects and enhances atherosclerosis ^39, 40^.

### Senescence-associated cellular secretory phenotype and inflammation

Senescent cells are characterized by short telomeres and other markers such as senescence-associated beta-glycosidase (SA-Beta Gal) p53, p21, and p16 ^ink4a^ ^41^. Experimental studies in murine models have shown the accumulation of senescent endothelial cells, macrophages, and smooth muscle cells in atherosclerotic plaques ^42^. Senescent cells express inflammatory cytokines in early stages of murine atherosclerosis and matrix-degrading enzymes in more advanced stages of atherosclerosis; both of these are implicated in atherogenesis and plaque instability ^32^. Depletion of these senescent cells reduces atherosclerosis and creates a more stable plaque composition, suggesting a causal role for senescent cells in inflammation, atherosclerosis, and plaque instability ^41^. These observations suggest that senolytic compounds, such as fisetin, that remove senescent cells may have athero-protective effects ^42^. Another important situation dealing with senescence and inflammation is chronic kidney disease. Uremia is typified by activation of innate immunity, which is characterized by activated monocytes and increased synthesis of pro-inflammatory cytokines (IL-6, tumor necrosis factor, and IL-1) ^43– 45^. In mice, chronic inflammation is related to cellular senescence, and senescent cells may upregulate and secrete pro-inflammatory cytokines as part of a senescence-associated secretory phenotype ^46^. This scenario is associated with progressive atherosclerosis and vascular calcification ^45^.

## Somatic hematopoetic mutations and inflammation

Aging is associated with accumulation of somatic hematopoetic mutations in certain genes that contribute to increased risk of hematological cancers and also to increased cardiovascular mortality ^47, 48^. This phenomenon is also called clonal hematopoetic mutations of indeterminate potential (CHIP). Mutations in *DNMT3A* or *TET2* genes, in particular, are associated with enhanced cardiovascular events ^47, 48^. Experimental observations in a murine model have demonstrated that enhanced atherosclerosis with *TET2* mutations is likely due to increased activity of NLRP3 inflammasome in monocytes ^47, 48^. Such age-dependent somatic mutations may contribute to increased inflammation and cardiovascular risk in the elderly.

## Impaired anti-inflammatory mechanisms

Acute inflammation generally resolves with time through the activity of a number of inflammation-resolving cellular and molecular mechanisms. These inflammation-resolving mechanisms involve lipid-derived pro-resolving humoral factors and cellular mechanisms. One of these cellular mechanisms involves clearance of apoptotic debris (efferocytosis) by macrophages mediated by several cellular receptors such as MerTK ^49– 51^. Thus, chronic persistent and smoldering inflammation may also result from failure of inflammation-resolving mechanisms in the host ^49– 51^. Molecules like CD47 that present a “don’t eat me” signal to the cells that clear apoptotic debris have been shown to be expressed in murine and human atherosclerotic plaques, and inhibition of CD47 stimulates efferocytosis and reduces atherosclerosis in murine models ^50^. Humoral mediators such as resolvins, protectins, and maresins are also involved in resolution of inflammation ^49^. Chronic inflammation likely results from an imbalance between pro-inflammatory and anti-inflammatory mediators; however, precise factors that modulate this delicate balance are poorly understood.

## Acute and chronic mental stress and inflammation

Mental stress is known to play an adverse role in cardiovascular disease, but the mechanisms linking stress to atherosclerosis have remained elusive. Recent studies on mice have shown that acute and chronic mental stress induce a pro-inflammatory response in which the brain sends signals to the bone marrow and spleen, stimulating hematopoiesis and production of pro-inflammatory monocytes (Ly6 ^hi^) that are recruited into atherosclerotic plaques, creating enhanced plaque inflammation ^52, 53^. Human studies have also provided support for the link between mental stress, inflammation, and atherosclerotic artery disease ^54– 58^. Interestingly, meditation has also been shown to reduce inflammatory markers and cardiovascular risk ^56^.

## Sleep deprivation and fragmentation, inflammation, and atherosclerosis

Sleep fragmentation or deprivation is associated with increased cardiovascular risk in human subjects ^59, 60^. Recent experimental studies in murine models have shown that sleep fragmentation enhances atherosclerosis by suppressing the release of hypocretin (orexin) from the hypothalamus; suppression of hypocretin results in increased myeloid hematopoiesis and production of pro-inflammatory monocytes, likely by stimulating the release of CSF-1 by pre-neutrophilic precursors in the bone marrow ^61^. Non-invasive imaging has shown an increased subclinical atherosclerotic burden in subjects with inadequate or fragmented sleep ^62^. Obstructive sleep apnea (OSA) is recognized as an independent risk factor for atherosclerotic cardiovascular disease ^63, 64^. In patients with OSA, pro-inflammatory molecules, such as soluble intercellular adhesion molecule 1, soluble vascular adhesion molecule 1, and MCP-1, were detected at very high levels with direct correlation to the desaturation index ^63, 64^. A study with a rat model of recurrent obstructive apneas reported increased leukocyte–endothelial cell interactions characterized by a significant increase in the flux of leukocyte rolling, number of rolling leukocytes, and number of adherent leukocytes ^65^.

## Summary and perspective

A large body of experimental and clinical observations highlights the role of inflammation in atherosclerosis and its complications (that is, plaque disruption and thrombosis). Some part of this inflammation is mediated through unhealthy lifestyles and conventional risk factors that can be addressed with aggressive lifestyle and risk factor modification. However, further incremental risk reduction may require agents that directly target inflammation. In keeping with these, a number of drugs targeting inflammation were tested in the clinic. The results, with the exception of those of the Canakinumab Anti-inflammatory Thrombosis Outcome Study (CANTOS), have largely been disappointing. Phospholipase inhibitors darapladib and varespladib, targeting pro-inflammatory phospholipases, failed to reduce cardiovascular events in randomized trials ^66, 67^. A mitogen-activated protein kinase inhibitor, losmapimod, also failed to reduce cardiovascular events ^68^. Because there appear to be multiple and redundant pathways for inflammation in athero-thrombosis, identification of the precise and optimal target for modification is relatively challenging. Recently, low-dose methotrexate was tested in the CIRT (cardiovascular inflammation reduction trial) and shown not to reduce cardiovascular events in high-risk patients ^69^. Interestingly, in this trial, low-dose Methotrexate also failed to reduce circulating inflammatory markers high-sensitivity C-reactive protein (hs-CRP) and IL-6 ^69^. In contrast, methotrexate has been shown to reduce inflammatory markers when used in the setting of high inflammatory burden ^7, 8, 70^. On the other hand, preliminary studies of cardiovascular benefits of low-dose colchicine have been encouraging, and large-scale randomized clinical trials examining the cardiovascular benefits of low-dose colchicine are ongoing ^71^. A recent significant development in this arena was the landmark CANTOS trial, in which canakinumab, a monoclonal antibody to IL-1β, showed significant cardiovascular benefit without changes in circulating lipids, albeit at the expense of an increase in fatal infections ^72^. High cost, increased risk of serious infection, and a relatively modest clinical benefit with canakinumab will make it unfeasible for routine clinical use.

It is clear that the search for agents that selectively target adverse vascular inflammation without interfering with beneficial aspects of inflammation must continue ^22, 34, 35^. Several promising avenues of research, including different strategies, are in active development. Among these are the blockage of CD40-induced tumor necrosis factor receptor-associated factor (TRAF) signaling in macrophages ^73^ and triggering receptor expressed on myeloid cells 1 (TREM-1) ^74^, strategies that activate the inhibitory immune receptor CD31 ^75^, or blocking CD47/SIRPA (signal regulatory protein alpha) signaling to promote inflammation resolution in plaques through enhanced efferocytosis ^50^. The CANTOS and CIRT trials showed important limitations related to immunosuppression. On the other hand, phospholipase inhibitors failed in reducing cardiovascular events. The optimal targets for modulation of inflammation need to be identified in order to develop anti-inflammatory therapies with high efficacy and safety. A potential and hopeful approach is the favorable modulation of atherosclerosis by vaccination by using antigens relevant to atherosclerosis. This would involve the development of antigen-specific antibodies or induction of antigen-specific Treg cells or other athero-protective immune responses. However, identification of the antigenic epitopes most relevant for atherosclerotic disease development in humans and some difficulties in vaccine design (for example, choice of the adjuvant, safety, and stability) are obstacles that will need to be overcome.
